# Late-onset pigment dispersion glaucoma after phakic implantable collamer lens implantation: A case report

**DOI:** 10.1097/MD.0000000000043225

**Published:** 2025-07-11

**Authors:** Zhengya Li, Cuicui Tang, Junling Yang, Mengyi Zhang, Renping Wu, Lianghong Peng

**Affiliations:** a Department of Ophthalmology, General Hospital of Southern Theater Command of PLA, Guangzhou, Guangdong Province, China.

**Keywords:** ICL implantation, late-onset complications, pigment dispersion glaucoma

## Abstract

**Rationale:**

Implantable collamer lens (ICL) implantation is a common refractive surgery for the treatment of high myopia, but its late-onset postoperative complications are rarely reported.

**Patient concerns:**

A 27-year-old patient presented with progressive vision loss and a visual field defect in the left eye, which had persisted for 1 year following ICL implantation 7 years prior. He was initially misdiagnosed with angle-closure glaucoma at a local hospital.

**Diagnoses:**

Upon detailed gonioscopy examination, we observed that the patient’s anterior chamber angle remained open, albeit with significant pigmentation present. Therefore, the diagnosis of pigment dispersion glaucoma should be revised.

**Interventions:**

To effectively manage intraocular pressure (IOP) and prevent progressive optic nerve damage in the left eye, we performed Kahook dual blade ab-interno trabeculectomy.

**Outcomes:**

The IOP of left eye ranged from 10 to 16 mm Hg 1 month after operation while 14 mm Hg at 1 year postoperatively without any antiglaucoma medication, in contrast to preoperative IOP levels that ranged from 25 to 29 mm Hg while 2 different antiglaucoma medications applied. Additionally, both visual acuity and visual field remained unchanged compared to preoperative.

**Lessons:**

The application of gonioscopy to accurately assess the angle status is crucial in the diagnosis and management of glaucoma. Furthermore, it is imperative to give due attention to potential late onset postoperative complications following ICL implantation.

## 1. Introduction

Implantable collamer lens (ICL) implantation is a widely performed refractive surgery, particularly in patients with high myopia.^[1]^ Common postoperative complications include concurrent cataract, loss of corneal endothelial cells, and secondary glaucoma which were rarely reported.^[2,3]^ This case report describes a 27-year-old patient who underwent bilateral ICL implantation 7 years ago. The patient developed decreased visual acuity and visual field defect in the left eye over 1 year with intraocular pressure (IOP) ranging from 25 to 29 mm Hg even if 2 antiglaucoma medicine applied. To manage IOP and prevent progressive optic nerve damage, we performed Kahook dual blade ab-interno trabeculectomy and obtained a satisfactory prognosis. The patient fully understood the purpose of this study and provided informed consent.

## 2. Case presentation

**Figure 1.**
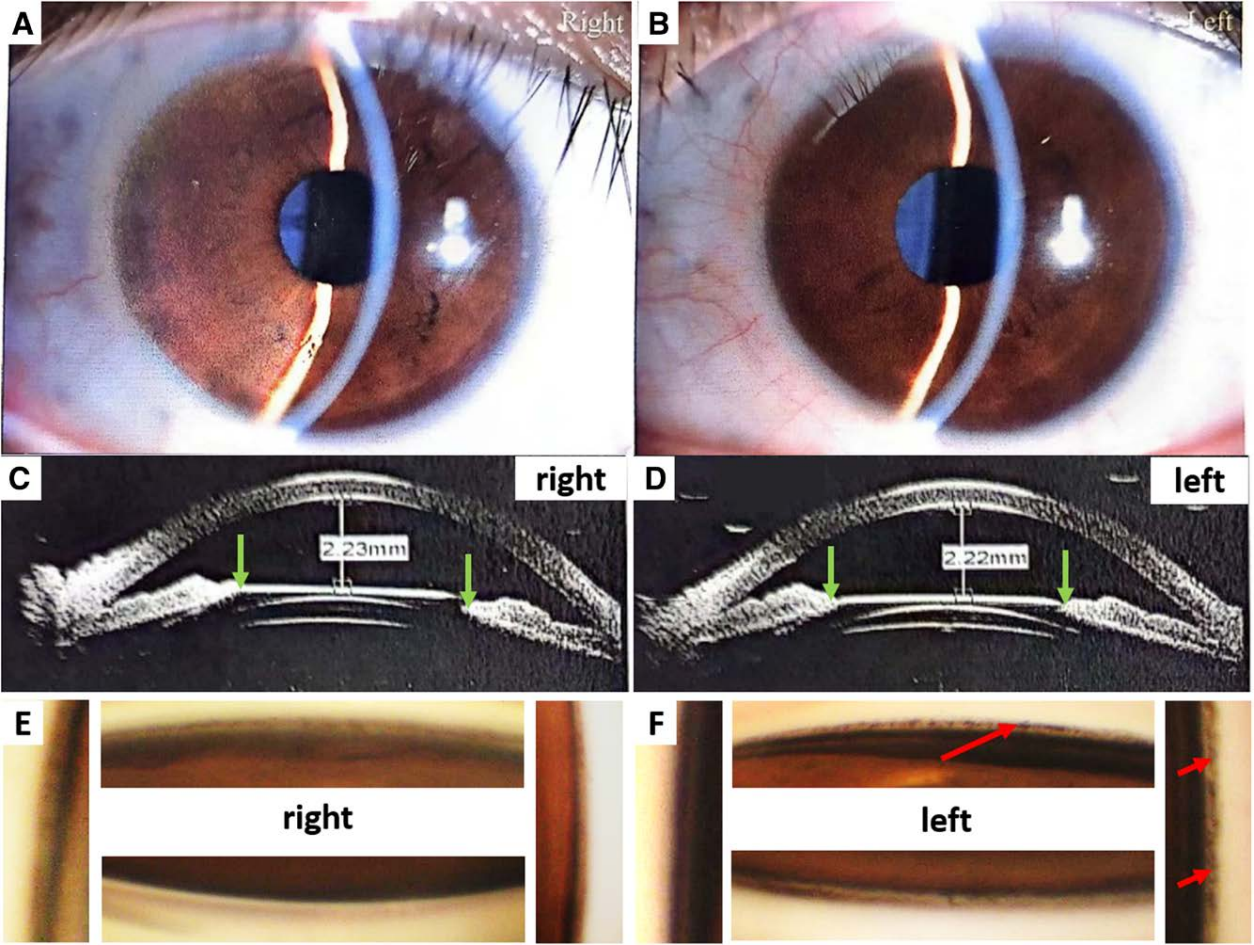
(A, B) Slit lamp examination in both eyes. The ICLs were positioned centrally with mid-hole fluid. (C, D) Ultrasound biomicroscopy (UBM) indicated direct contact between the ICL and the posterior surface of the irises in both eyes (*green arrows*) with relatively high vaults. (E, F) Gonioscopy examination revealed significant pigmentation on the trabecular meshwork while the anterior chamber angle open, especially in the inferior and nasal quadrants of the left eye (*red arrows*). ICL = implantable collamer lens.

**Figure 2.**
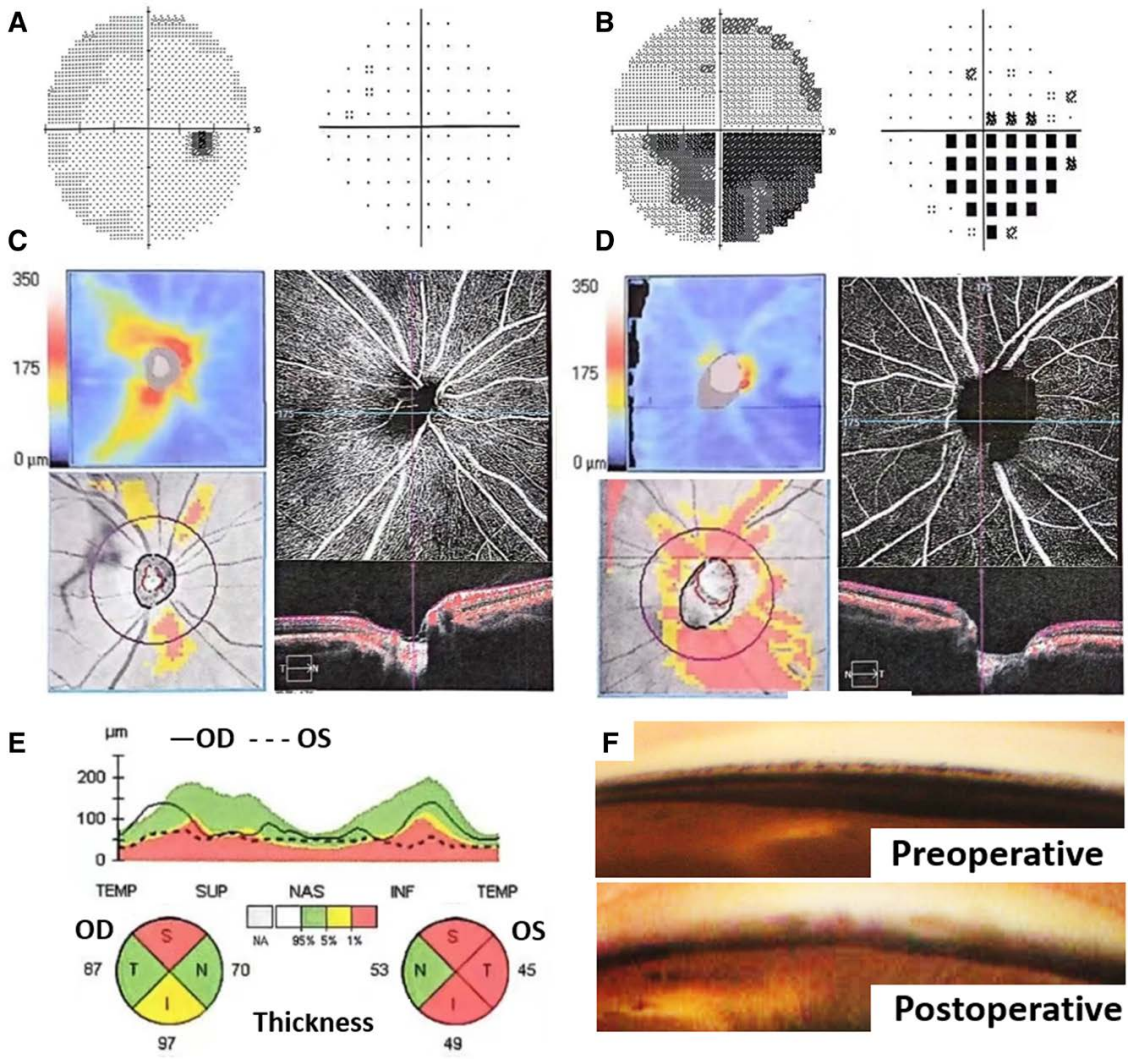
(A, C, E) The right eye exhibited normal visual field and optic nerve OCT performance. (B, D, E) Visual field defect of temporal inferior quadrant consistent with optic nerve thickness thinning and reduced vessel density was observed in the left eye. (F) Postoperative gonioscopy of the left eye revealed a “clean-up” chamber angle of inferior quadrant in contrast to preoperative.

A 27-year-old male patient, who underwent bilateral ICL implantation 7 years ago due to high myopia (−11.5D preoperatively), presented with progressive vision loss and a visual field defect in the left eye at a local hospital 1 month prior. He was diagnosed with angle-closure glaucoma in the left eye and was advised to consider either ICL removal or iris laser perforation to alleviate potential pupil block. Upon initial examination at our hospital, the visual acuity of the right eye was 20/20, whereas that of the left eye was 20/40, which could not be corrected. Goldmann applanation tonometry revealed IOP at 16 mm Hg in the right eye while 25 mm Hg in the left eye. Slit lamp examination revealed transparent corneas in both eyes, and the ICL were positioned centrally with a mid-hole fluid (Fig. 1A, B). Ultrasound biomicroscopy (UBM) indicated direct contact between the ICL and posterior surface of the iris in both eyes (Fig. 1C, D), accompanied by relatively high vaults, leading to shallow anterior chamber depths. This may have contributed to the misdiagnosis of angle-closure glaucoma during his initial visit to the local hospital. To further assess the status of the anterior chamber angle, we performed gonioscopy, which demonstrated that although the angle remained open, significant pigmentation was observed particularly in the inferior and nasal quadrants trabecular meshwork of the left eye (Fig. 1E, F). Additionally, a temporal inferior visual field defect (Fig. 2B) consistent with diffuse optic nerve thinning and decreased vessel density was noted in the left eye (Fig. 2D, E), in contrast to the normal results for the right eye (Fig. 2A, C, E). Consequently, we revised our diagnosis to secondary pigment dispersion glaucoma in the left eye. The patient had been prescribed 2 antiglaucoma medications for his left eye at local hospital: brinzolamide twice daily and tafluprost once per night. However, these treatments yielded limited efficacy with persistently elevated IOP ranging from 25 to 29 mm Hg.

Considering that the removal of the ICL may lead to a significant decrease in uncorrected visual acuity which against the patient’s wish, we performed Kahook dual blade ab-interno trabeculectomy (4–8 o’clock) for the patient’s left eye to manage IOP and prevent progressive optic nerve damage. This procedure effectively addressed the obstruction of aqueous humor drainage caused by trabecular pigmentation with minimizing surgical trauma.^[4]^ Fortunately, 1 week after the operation, the IOP of the left eye decreased to 11 mm Hg, fluctuating between 8 and 12 mm Hg over 24-hour without any antiglaucoma medication. Gonioscopy of the goniotomy site revealed a ‘clean-up’ chamber angle (Fig. 2F). 1 month after operation, the left IOP stabilized from 10 to 16 mm Hg without pharmacological intervention. To confirm the long-term success of the operation, we conducted an additional follow-up examination at about 1 year postoperative (Fig. S1, Supplemental Digital Content, https://links.lww.com/MD/P359). The findings revealed that the IOP of left eye was 14 mm Hg still without medicine help, and the visual field defects remained almost unchanged compared to preoperative assessments. Gonioscopy demonstrated faintly visible traces of the trabeculotomy incision at 4 to 8 o’clock directions, while UBM clearly visualized the trabecular meshwork incision at 6 o’clock. These observations further support the long-term efficacy and stability of the operation.

## 3. Discussion

In this case, we report a patient with pigment-disseminated glaucoma 7 years after ICL implantation, for whom we performed Kahook dual blade ab-interno trabeculectomy. Postoperatively, the patient achieved stable IOP control, exhibited no significant progression in visual field defects, required no long-term use of antiglaucoma medications and successfully avoided ICL removal.

Common complications after ICL implantation include anterior subcapsular cataract, loss of corneal endothelial cells, and secondary glaucoma.^[2,3]^ However, secondary glaucoma has rarely been reported.^[5]^ Sanchez-Galeana et al reported a case of pigment dispersion syndrome in a patient who had previously undergone ICL implantation.^[6]^ 6 months after surgery, the IOP in the affected eye showed a sustained overall increase, and ICL removal with trabeculectomy was managed with significantly decreased visual acuity. A similar case of elevated IOP in 1 eye was reported by Chung et al.^[7]^ In this case, despite the postoperative ICL vault being normal, significant pigment deposition on the ICL surface was observed. Additionally, the long-term use of antiglaucoma medications appears to be unavoidable. Notably, 1 case of late-onset pigment dispersion glaucoma occurred 8 years after ICL surgery, resulting in visual impairment in the left eye.^[8]^ Ultimately, the ICL in this eye was removed, leading to a significant decrease in vision. These cases suggested that ICL implantation may result in structural alterations in the iridocorneal angle and increased pigmentation of the trabecular meshwork, potentially leading to elevation of IOP and, in some instances, secondary glaucoma.

In this case, UBM revealed a narrow iridocorneal angle, which led to a misdiagnosis of angle-closure glaucoma during the patient’s initial visit to the local ophthalmologist. However, upon gonioscopy examination, we discovered significant pigmentation obstructing the trabecular meshwork, particularly in the left eye. We propose that the pigmentation etiology is the direct mechanical friction between the ICL surface and the posterior surface of the iris.

Kahook dual blade ab-interno trabeculectomy^[4,9]^ represents the optimal surgical option for this case. This procedure effectively opens the aqueous humor drainage by directly incising the trabecular meshwork with minimal surgical trauma, thereby avoiding the decline in uncorrected visual acuity that would otherwise result from ICL removal.

## 4. Conclusion

The utilization of gonioscopy to accurately assess the status of the anterior chamber angle in glaucoma diagnosis is crucial. In addition, sufficient attention should be paid to late-onset complications after ICL implantation. Kahook dual blade ab-interno trabeculectomy might provide an optimal prognosis for pigment dispersion glaucoma.

## Author contributions

**Conceptualization:** Zhengya Li, Lianghong Peng.

**Data curation:** Cuicui Tang, Junling Yang, Mengyi Zhang, Renping Wu.

**Funding acquisition:** Zhengya Li, Lianghong Peng.

**Investigation:** Lianghong Peng.

**Writing – original draft:** Cuicui Tang.

**Writing – review & editing:** Zhengya Li.

## Supplementary Material

SUPPLEMENTARY MATERIAL
