# Supplementary material for: Late-onset pigment dispersion glaucoma after phakic implantable collamer lens implantation: A case report
Source: Medicine (Baltimore). 2025 Jul 11;104(28):e43225. doi: 10.1097/MD.0000000000043225 (PMC12263071; doi:10.1097/MD.0000000000043225)
Supplement: SUPPLEMENTARY MATERIAL [file medi-104-e43225-s001.docx]

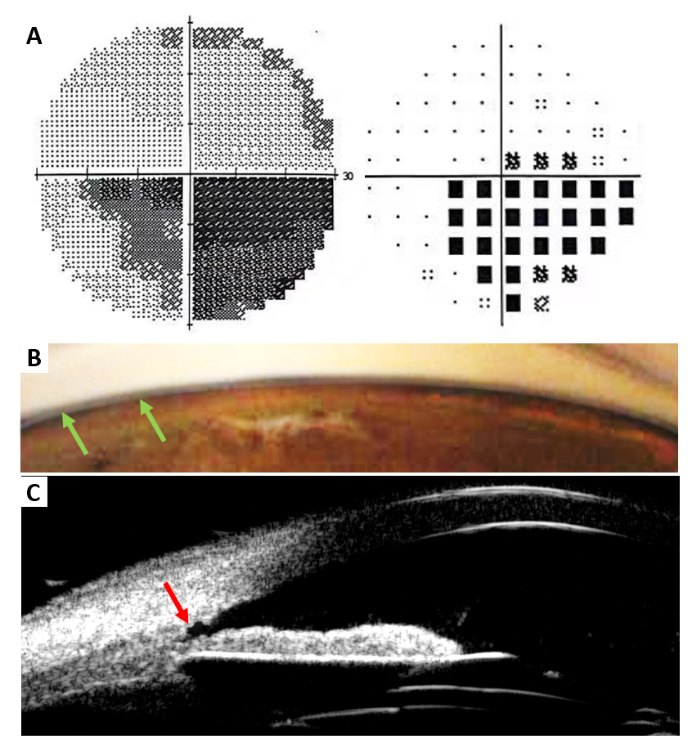
**Supplementary Fig.S1 (A)** The visual field of left eye showed a temporal inferior quadrant defect. (B) Gonioscopy demonstrated faintly visible traces of the trabeculotomy incision at 4–8 o’clock directions (*green arrows*). (C) UBM visualized the trabecular meshwork incision at 6 o’clock (*red arrow*).
